# Supplementary material for: Identifying Stabilin-1 and Stabilin-2 Double Knockouts in Reproduction and Placentation: A Descriptive Study
Source: Int J Mol Sci. 2020 Sep 30;21(19):7235. doi: 10.3390/ijms21197235 (PMC7583024; doi:10.3390/ijms21197235)
Supplement: Supplementary file 1 [file ijms-21-07235-s001.pdf]

Identifying Stabilin-1 and Stabilin-2 Double Knockouts in Reproduction and Placentation: A Descriptive Study

Soon-Young Kim [1,2], Eun-Hye Lee [1], Eun Na Kim [3], Woo-Chan Son [3], Yeo Hyang Kim [4,5], Seung-Yoon Park [6], In-San Kim [7,8] and Jung-Eun Kim [1,2,*]

* Correspondence: kjeun@knu.ac.kr

Supplementary Materials

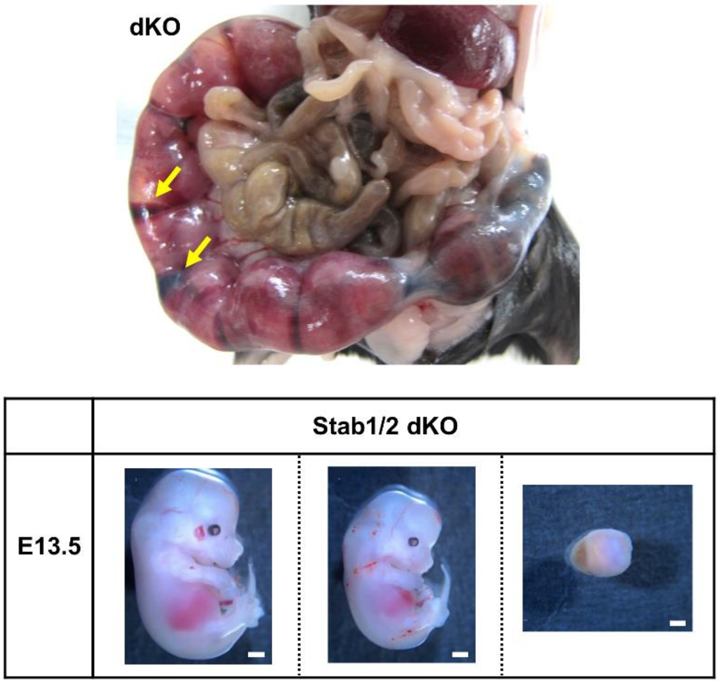

Figure S1. Uterine horns with E13.5 embryos in pregnant Stab1/2 dKO mice. Yellow arrows in Stab1/2 dKO indicate blood within uterine horns. High-magnification images of unevenly sized embryos or undefined tissue at E 13.5. Scale bar, 1 mm

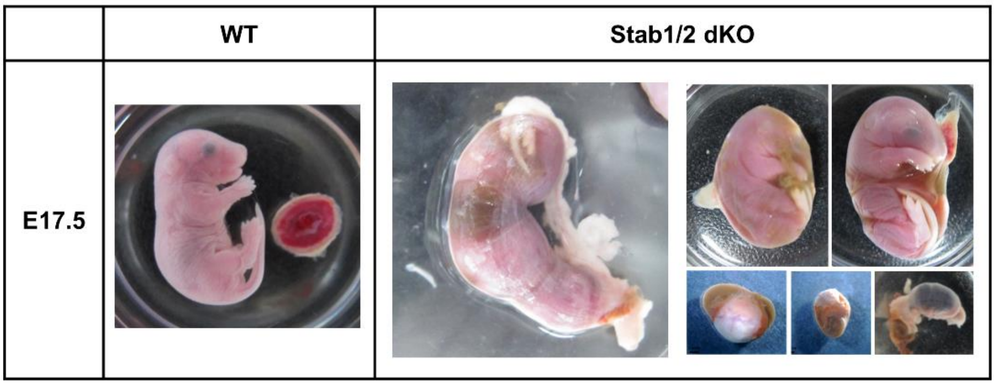

Figure S2. Images of unevenly sized embryos or undefined tissue in pregnant Stab1/2 dKO mice compared to normal-sized embryo and placenta of pregnant WT mice at E17.5.

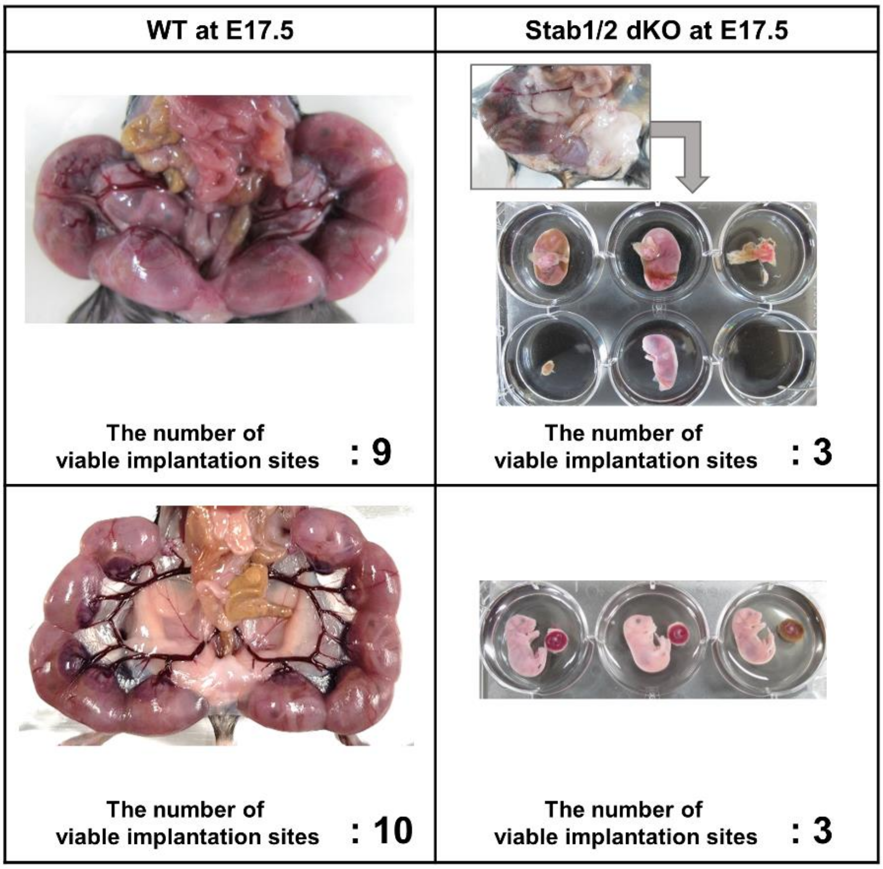

Figure S3. Images of the uteri or E17.5 embryos in WT and Stab1/2 dKO females and the number of viable implantation sites

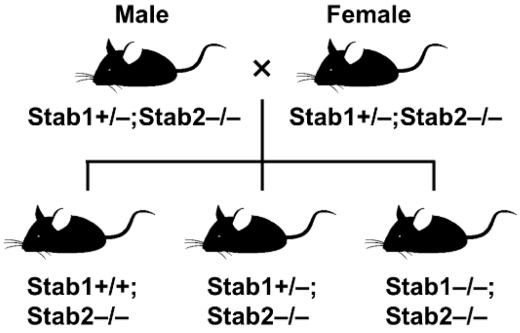

Genotype of Pups	Number
Stab1+/+;Stab2–/–	11
Stab1+/–;Stab2–/–	47
Stab1–/–;Stab2–/–	3

Figure S4. Pedigree and the number of pups generated by crossing between male and female mice with Stab1 heterozygous and Stab2 knockout genotypes. WT, +/+; heterozygous, +/-; knockout, -/-
